# Changing Etiological Spectrum of Premature Ovarian Insufficiency over the Past Decades: A Comparative Analysis of Two Cohorts from a Single Center

**DOI:** 10.3390/diagnostics15131724

**Published:** 2025-07-06

**Authors:** Szilvia Csehely, Adrienn Kun, Edina Orbán, Tamás Katona, Mónika Orosz, Zoárd Tibor Krasznai, Tamás Deli, Attila Jakab

**Affiliations:** 1 Department of Obstetrics and Gynaecology, Faculty of Medicine, University of Debrecen, Nagyerdei krt. 98, 4032 Debrecen, Hungary; kun.adrienn@med.unideb.hu (A.K.); orban.edina@med.unideb.hu (E.O.); orosz.monika@med.unideb.hu (M.O.); krasznai.zoard@med.unideb.hu (Z.T.K.); deli.tamas@med.unideb.hu (T.D.); 2 Doctoral School of Informatics, University of Debrecen, 4028 Debrecen, Hungary; hello@katonatomi.com; 3 Department of Data Science and Visualization, Faculty of Informatics, University of Debrecen, 4028 Debrecen, Hungary

**Keywords:** premature ovarian insufficiency, etiology of premature ovarian insufficiency, iatrogenic POI

## Abstract

**Background**: Premature ovarian insufficiency (POI) is a complex and heterogeneous condition affecting women of reproductive age. Historically, most POI cases have been classified as idiopathic due to limited diagnostic capabilities. However, due to the success of oncologic treatments and the increasing number of gynecologic surgeries enabled by improved diagnostics, the proportion of iatrogenic POI cases has risen substantially. **Objectives**: To investigate the current prevalence of POI etiologies, to compare the etiological distribution between two POI cohorts from a single tertiary center—one historical (1978–2003) and one contemporary (2017–2024)—and to explore how the spectrum of underlying causes has changed over the past four decades. **Methods**: Data from 111 women diagnosed with POI between 2017 and 2024 were retrospectively reviewed and compared with those from a historical cohort of 172 patients. Etiologies were classified as genetic, autoimmune, iatrogenic, or idiopathic. Statistical comparisons were performed using chi-square and z-tests. Hormonal profiles and reproductive outcomes were also analyzed. **Results**: The current prevalence of POI etiologies is as follows: genetic 9.9%, autoimmune 18.9%, iatrogenic 34.2%, idiopathic 36.9%. In the historical POI cohort, etiologies were classified as genetic in 11.6%, autoimmune in 8.7%, iatrogenic in 7.6%, and idiopathic in 72.1%. The changes in the prevalence of autoimmune, iatrogenic, and idiopathic POI were statistically significant (*p* < 0.05). Reproductive outcomes remained limited: 10 pregnancies occurred in each cohort, with 7 live births in the contemporary group. **Conclusions**: Our findings suggest a significant shift in the etiological landscape of POI, with a notable, more than fourfold rise in identifiable iatrogenic cases and a twofold increase in the autoimmune group, resulting in a halving of idiopathic POI. Prevalence of genetic etiology remained unchanged. While diagnostic capabilities have improved, reproductive outcomes remain largely unchanged and suboptimal.

## 1. Introduction

Primary ovarian insufficiency (POI) is a condition defined by the cessation of normal ovarian function before the age of 40, characterized by persistent menstrual irregularities (amenorrhea or oligomenorrhea for at least four months) and elevated serum FSH levels (>25 U/L). This definition aligns with the criteria recently established by the European Society of Human Reproduction and Embryology (ESHRE) [1]. Earlier criteria suggested a higher FSH threshold [2] and required two separate measurements at least four weeks apart. Existing data suggest that POI affects at least 1% of women under 40, with an estimated prevalence of 1 in 10,000 among women aged 15–29 and approximately 1 in 1000 among those aged 30–39 [3]. More recent meta-analyses (2019, 2023) have provided refined prevalence estimates by country and ethnicity, reporting similar overall rates of 3.5% (95% CI 3.0–4.0%) and 3.7% (95% CI 3.1–4.3%), respectively [4,5]. Notably, these analyses highlighted geographic differences, with higher prevalence observed in North America compared to Europe [5].

POI adversely affects women’s health both in the short and long term. In the short term, it may present with infertility, menstrual disturbances, vasomotor symptoms, mood changes, and decreased quality of life [3]. In the long term, women with POI face increased risks of osteoporosis, cardiovascular disease [6], cognitive decline, and premature mortality due to hypoestrogenism [3].

POI has a multifactorial etiological background, encompassing genetic abnormalities, autoimmune disorders, and induced (iatrogenic or environmental) damage to the ovarian follicular reserve. However, despite extensive diagnostic evaluation, the underlying cause remains unidentified in up to 50% of cases, leading to classification as idiopathic POI [1].

### 1.1. Genetic Causes of POI

Chromosomal abnormalities have been identified in approximately 12–13% of POI cases, primarily involving the X chromosome. These abnormalities are more frequently observed in women with primary amenorrhea (21.4%) compared to those with secondary amenorrhea (10.6%) [7,8]. Among X-linked chromosomal abnormalities, Turner syndrome (45,X and mosaic variants: 45,X/46,XX; 45,X/47,XXX; 45,X/46,XY; 45,X/47,XYY; and 46,X,del(Xq) [9]) is the most commonly diagnosed, affecting approximately 1 in 2000 to 2500 live-born females [10], leading to accelerated follicular atresia due to partial or complete loss of one X chromosome. Another relatively common X-linked cause of POI is the fragile X premutation involving the FMR1 gene. Women carrying 55–200 CGG repeats in the FMR1 gene are classified as premutation carriers [3]. Among these carriers, approximately 20–30% develop fragile X–associated primary ovarian insufficiency (FXPOI) [11], a significantly higher prevalence compared to about 3.5% in the general population [1,4,5]. The risk of developing POI is strongly influenced by CGG repeat size, with women carrying 70–100 repeats at the highest risk [11], reflecting a non-linear relationship (Sherman paradox [12]); in contrast, full mutations (>200 repeats), associated with fragile X syndrome, are not linked to elevated risk of POI [11,13]. In terms of familial patterns, research indicates that approximately 11.5% of familial POI cases and 3.2% of sporadic cases harbor FMR1 premutations, highlighting the importance of family history in assessing risk [14,15].

POI is a highly heterogeneous disorder involving mutations in more than 75 genes, primarily linked to meiosis and DNA repair, though most cases still lack a clear genetic diagnosis [16]. Mutations in genes such as BMP15, GDF9, NOBOX, FSHR, LHR, FOXL2, and CYP19A1 have been implicated in POI [3,17]. Certain syndromic conditions, including Perrault syndrome, Bloom syndrome, and Ataxia–telangiectasia, also feature POI as part of their clinical presentation [3].

### 1.2. Autoimmune POI

Autoimmune mechanisms are implicated in approximately 4–30% of spontaneous POI cases. Conditions such as Hashimoto’s thyroiditis, Addison’s disease, Graves’ diseases, myasthenia gravis, type 1 diabetes mellitus, rheumatoid arthritis, systemic lupus erythematosus (SLE), inflammatory bowel disease (IBD), vasculitis, sarcoidosis, Sjögren’s syndrome, psoriasis, vitiligo, and celiac disease may be associated with POI [3,18,19,20]. Hashimoto’s thyroiditis is notably common in women with POI, conferring an 89% higher risk of amenorrhea and a 2.4-fold increased risk of infertility due to ovarian failure compared to non-affected individuals according to a recent Taiwanese cohort study [21]. Furthermore, the presence of thyroid autoantibodies (TgAb, TPOAb) has been associated with an increased risk of POI, even in women with normal thyroid function [22]. Autoimmune oophoritis, characterized by lymphocytic infiltration selectively targeting steroidogenic cells, leads to progressive follicular depletion. The detection of steroidogenic cell autoantibodies, particularly against 21-hydroxylase, supports the autoimmune etiology of POI [3,18,19].

### 1.3. Infectious Causes

Infectious causes of POI are rare but may include mumps [23], human immunodeficiency virus (HIV) infection [4,5], tuberculosis, malaria, cytomegalovirus (CMV), varicella [3,24] and, more recently, SARS-CoV-2 (COVID-19) infection [25]. While earlier research suggested a possible link between HIV infection and earlier menopause [5], more recent data have not consistently confirmed a significant difference in menopausal age [26]. Several case reports have documented the onset of POI in women shortly following SARS-CoV-2 infection [27,28,29]. However, the long-term reproductive effects remain uncertain and warrant further investigation [25,30].

### 1.4. Toxic Causes

A systematic review of 97 eligible studies identified phthalates, bisphenol A, pesticides, and tobacco as major environmental pollutants negatively affecting ovarian function. These exposures, occurring from prenatal life to adulthood, were associated with increased follicular atresia and accelerated ovarian aging, potentially leading to an earlier onset of menopause [31]. Among toxic environmental factors, bisphenol compounds—including BPA and its structural analogs BPS, BPF, and BPB—have been increasingly implicated in ovarian toxicity, with growing evidence suggesting that these endocrine disruptors may impair folliculogenesis and contribute to the risk of POI [32,33,34,35,36]. Smoking has been consistently linked to an increased risk of premature ovarian insufficiency, with both cohort studies and meta-analyses showing a dose-dependent association [37] and up to 2.75-fold elevated risk among smokers [23].

### 1.5. Metabolic Causes

Classic galactosemia is a rare autosomal recessive metabolic disorder caused by a deficiency of galactose-1-phosphate uridyltransferase (GALT) [38], leading to toxic accumulation of galactose and its metabolites in organs with high GALT expression, including the ovaries [39]. Several mechanism have been postulated to explain POI in patients with galactosemia, including potential oocyte toxicity, impaired FSH signaling due to aberrant glycosylation, and epigenetic dysregulation; however, the precise pathophysiology and timing of ovarian damage remain unclear [38,39,40]. Importantly, not all those affected develop POI, and some retain ovarian function or achieve spontaneous pregnancy [41].

### 1.6. Iatrogenic Etiology

According to data from the Childhood Cancer Survivor Study (CCSS), the prevalence of POI is significantly higher among childhood cancer survivors compared to the general population. Findings from both the CCSS and the St. Jude Lifetime Cohort (SJLIFE) studies showed that POI prevalence among survivors aged 21 to 40 years increased from 7.9% to 18.6% in the CCSS cohort, and from 7.3% to 14.9% in the SJLIFE cohort [42].

Chemotherapy represents a significant risk factor for the development of premature ovarian insufficiency (POI). Among chemotherapeutic agents, alkylating compounds such as cyclophosphamide and platinum-based drugs like cisplatin are considered the most gonadotoxic. The depletion of ovarian follicles occurs through mechanisms involving direct DNA damage, oxidative stress, and mitochondrial dysfunction [43,44].

Radiotherapy poses a substantial risk for POI, as even low doses (2 Gy) can destroy half of the ovarian follicle pool, while whole pelvic irradiation at curative doses (20–30 Gy) may result in ovarian failure rates as high as 97%, with gonadotoxicity influenced by factors such as patient age, baseline ovarian reserve, radiation field, and total dose [45].

Iatrogenic POI frequently follows ovarian surgeries [7], with numerous studies reporting that laparoscopic cystectomy for endometriomas is associated with a greater risk of ovarian damage [46,47] and more pronounced declines in serum AMH levels compared to similar procedures for dermoid cysts [48]. Supporting this, a large population-based study of more than 279,000 women reported that individuals with endometriosis had a sevenfold increased risk of undergoing surgical menopause, which occurred on average 1.6 years earlier than in women without endometriosis. Moreover, they showed significantly higher odds of premature surgical menopause before age 40 (OR 2.11) and an increased risk of spontaneous POI (OR 1.36) [49].

### 1.7. Study Objective

It is widely speculated that the reported increase in POI prevalence is multifactorial, partly attributable to greater medical awareness leading to more frequent diagnosis, and partly to unfavorable shifts in underlying etiological factors. However, despite intensive research on POI, the relative contribution of these factors to the rising prevalence remains uncertain.

In this study, we compared the etiological distribution of premature ovarian insufficiency (POI) between a historical cohort (1978–2003), previously published in 2004, and a contemporary cohort (2017–2024), with a particular focus on identifying shifts in the prevalence of genetic, autoimmune, iatrogenic, and idiopathic causes. This comparison was prompted by the availability of detailed archived data from an earlier institutional cohort, providing a unique opportunity to assess temporal trends and highlight the clinical implications of evolving diagnostic practices.

## 2. Patients and Methods

In this retrospective comparative study of two cohorts, we performed a detailed etiological analysis of primary ovarian insufficiency (POI) over an eight-year period (2017–2024) at the Gynecological Endocrinology and Menopause Unit of our university hospital (University of Debrecen, Hungary) (contemporary cohort, Group 1). We then compared these findings with data from a previously published analysis conducted at the same institution. The contemporary cohort included a total of 111 women (mean age 32.8 ± 8.8 years) diagnosed with POI between January 2017 and June 2024 (Group 1). To assess temporal changes in the distribution of POI etiologies, this group was compared with a historical cohort of POI patients diagnosed between 1978 and 2003, as reported in a previous publication [50]. This historical cohort (Group 2) was selected based on comparable diagnostic criteria, sample size, and methodological consistency.

The spectrum of laboratory examinations applied in POI diagnosis did not differ between the two study periods; however, the diagnostic criteria have been modified. In the publication of the historical cohort (Group 2), based on the former recommendations, an FSH threshold of >40 U/L was used to diagnose POI (formerly referred to as premature ovarian failure, POF), confirmed by two separate measurements taken at least four weeks apart [51]. In contrast, in the contemporary cohort, POI was diagnosed according to the NICE guideline NG23 criteria (2015, reviewed 2019) [2], which require menstrual irregularities (≥4 months of amenorrhea or oligomenorrhea) and elevated serum FSH levels (>30 U/L), confirmed by two separate measurements at least four weeks apart in women under the age of 40.

### 2.1. Data Collection and Etiological Classification

Patient data from Group 1 were retrospectively extracted from the electronic hospital database. Detailed demographic information, as well as medical, gynecological, surgical, and oncological histories, gravidity, and parity, were collected. Patient data from Group 2 were retrieved from the original publication (2004) and, where available, from archived patient files.

A detailed general medical, obstetric, gynecologic, and menstrual history was recorded, including the date of the last menstruation. Pregnancy was excluded. Baseline laboratory assessments included measurements of serum follicle-stimulating hormone (FSH), luteinizing hormone (LH), 17β-estradiol (E2), thyroid-stimulating hormone (TSH), triiodothyronine (T3), and thyroxine (T4). Autoimmune screening involved testing for anti-thyroid peroxidase (anti-TPO) antibodies, anti-thyroglobulin (anti-Tg) antibodies, and adrenal cortex (21-hydroxylase) antibodies to identify potential autoimmune etiologies. Due to the retrospective nature of the study, not all laboratory data were consistently available for every patient.

Genetic investigations—including conventional karyotyping and *FMR1* premutation analysis—were selectively performed based on clinical judgment, particularly in cases without an evident iatrogenic cause or where clinical features suggested a possible genetic origin. Genetic testing was generally omitted in patients with a clear iatrogenic etiology, such as chemotherapy, radiotherapy, or surgical bilateral oophorectomy.

In terms of etiology, patients were categorized into four main groups:

- Genetic POI: Including cases with chromosomal abnormalities (e.g., Turner syndrome, triple X syndrome) or *FMR1* premutation.
- Autoimmune POI: Defined by the presence of adrenal cortex antibodies (21OH-Ab), clinically confirmed Hashimoto’s thyroiditis with anti-TPO and/or anti-thyroglobulin (Tg) positivity, or other established autoimmune diseases (e.g., Addison’s disease, systemic lupus erythematosus, Graves’ disease). The classification of autoimmune POI cases in our study was based on the 2016 ESHRE guideline recommendations, which at that time supported the inclusion of thyroid autoimmunity in the diagnostic evaluation [52].
- Iatrogenic POI: Secondary to chemotherapy, radiotherapy, or surgical ovarian damage (e.g., oophorectomy, extensive cystectomy, adnexectomy).
- Idiopathic POI: No identifiable cause despite comprehensive evaluation.

### 2.2. Statistical Analysis

Patients’ data were stored and managed in a PostgreSQL 16.2 database to ensure reliable and structured data handling. For statistical analysis, we used Python 3.12 with libraries such as pandas for data manipulation, NumPy 2.2.0 for numerical operations, and statsmodels 0.14.4 [53] for logistic regression modeling. We also applied statistical tests including chi-square test, Z-test, and two-sample *t*-test to assess differences between groups.

## 3. Results

### 3.1. Patient Characteristics and Prevalence of POI Etiologies in Group 1 (Contemporary Cohort)

A total of 111 patients diagnosed with POI and complete etiological data were included in the analysis. Among them, 84.7% (n = 94) were diagnosed with secondary amenorrhea, and 15.3% (n = 17) with primary amenorrhea. Symptoms of POI began before the age of 30 in 40.5% of cases (n = 45), while 59.5% (n = 66) experienced symptom onset between the ages of 30 and 40. Patients characteristics are summarized in Table 1.

**Figure 1:**
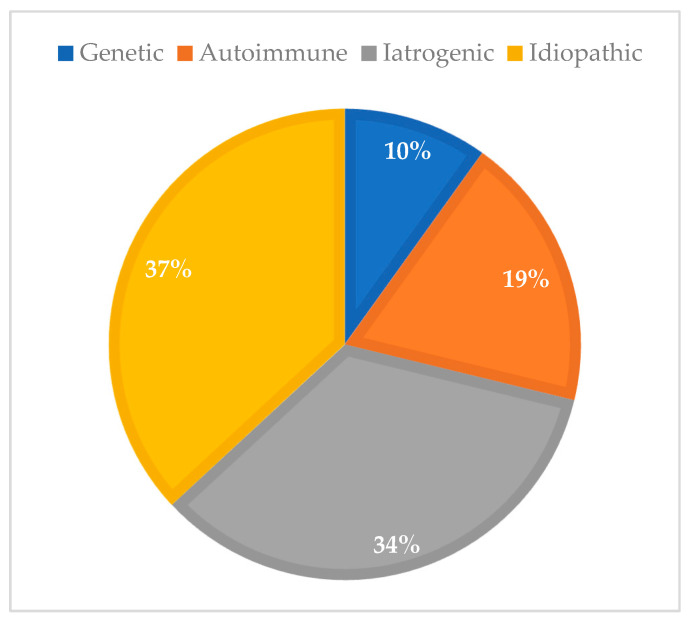
Prevalence of POI etiologies in Group 1 (contemporary cohort, 2017–2024; n = 111).

Regarding etiology, among POI patients 36.9% were classified as idiopathic, 34.3% as iatrogenic, 9.9% as genetic, and 18.9% as autoimmune (Figure 1).

### 3.2. Patient Characteristics and Prevalence of POI Etiologies in Group 2 (Historical Cohort)

In a historical cohort from 1978 to 2003 including 172 patients diagnosed with POI, complete etiological data were retrieved from a previously published analysis by our center [50]. The mean age of the patients was 30.17 ± 5 years. Among them, 89.5% (n = 154) were diagnosed with secondary amenorrhea, and 10.5% (n = 18) with primary amenorrhea. POI manifested before the age of 30 in 48.3% of cases (n = 83), while 51.7% (n = 88) experienced symptom onset between the ages of 30 and 40.

**Figure 2:**
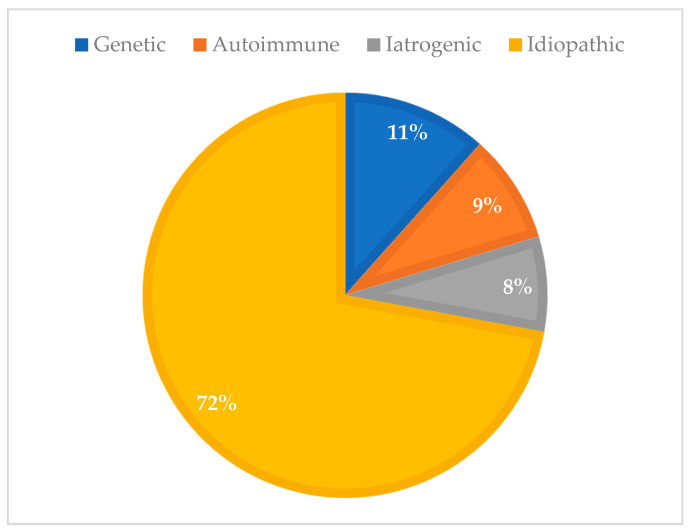
Prevalence of POI etiologies in Group 2 (historical cohort, 1978–2003; n = 172).

The distribution of POI etiologies was as follows: idiopathic in 124 cases (72.1%), genetic (including gonadal dysgenesis) in 20 cases (11.6%), iatrogenic in 13 cases (7.6%), and autoimmune in 15 cases (8.7%) (Figure 2)

### 3.3. Comparative Analysis of Group 1 (Contemporary Cohort) and Group 2 (Historical Cohort)

The detection rate of POI differed between the two groups, showing a significant increase from 6.6 patients per year in the historical cohort to 13.9 patients per year in the contemporary cohort (rate ratio = 2.10, 95% CI: 1.65–2.66, *p* < 0.0001), as determined by Poisson rate comparison, representing a 2.1-fold increase. Neither the age at POI manifestation nor the distribution of patients below and above 30 years of age differed significantly between the two cohorts.

**Figure 3:**
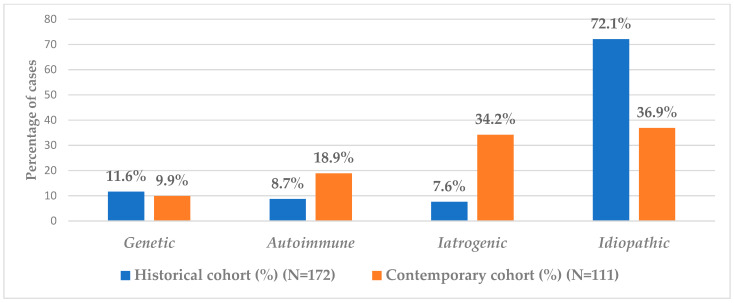
Comparison of POI etiologies in the historical and contemporary cohort.

The comparison between historical and contemporary cohorts revealed significant changes in the prevalence of POI etiologies, with the exception of genetic etiology (Figure 3). The most pronounced increase was observed in the proportion of iatrogenic POI cases. While only 7.6% (13/172) of POI cases in the 1978–2003 cohort were classified as iatrogenic, this proportion rose to 34.2% (38/111) in the current cohort (Figure 3). This difference was statistically significant, as confirmed by both Z-test (z = −5.70, *p* < 0.0001) and chi-square analysis (χ^2^ = 30.71, *p* < 0.0001). To further explore the temporal association, a binary logistic regression model was applied, using the diagnostic period (1978–2003 vs. 2017–2024) as the independent variable. Being diagnosed between 2017 and 2024 was significantly associated with higher odds of iatrogenic POI (OR = 6.37, 95% CI: 3.20–12.67, *p* < 0.0001), suggesting an etiologic shift potentially linked to changes in clinical practice, including more frequent surgical interventions and improved oncological outcomes.

The rate of autoimmune POI cases rose from 8.7% (15/172) in the historical cohort to 18.9% (21/111) in the contemporary cohort (Figure 3). This increase was statistically significant, as confirmed by both the Z-test (z = −2.51, *p* = 0.012) and the chi-square test (χ^2^ = 5.43, *p* = 0.020). It is important to note that both cohorts included patients with Hashimoto’s thyroiditis, which may partially explain the higher proportion compared to studies applying stricter autoimmune criteria. The proportion of idiopathic POI cases was significantly lower in the contemporary cohort (36.9%, 41/111) compared to the historical cohort (72.1%, 124/172) (Figure 3). Both the Z-test (z = 5.86, *p* < 0.0001) and the chi-square test (χ^2^ = 32.87, *p* < 0.0001) confirmed a statistically significant reduction, indicating a notable shift in the etiologic distribution of POI over time.

### 3.4. Detailed Comparative Analysis of Genetic, Autoimmune, and Iatrogenic Background of POI in the Two Cohorts

In the historical cohort, 20 patients (11%) were classified as having a genetic etiology, of whom 15 were diagnosed with Turner syndrome. The reported karyotypes included 45,X0, 45,X0/46,XX mosaicism, 45,X0/46,XXq21, and 46,XX with Xq deletion. In four additional cases, gonadal dysgenesis was diagnosed without a definitive syndromic classification. In the contemporary cohort, 11 patients (10%) were identified with a genetic etiology. The majority were diagnosed with Turner syndrome, with the following karyotypes reported: 45,X0; 45,X0/46,XX; 46,X,i(Xq)(15); and 46,X,del(Xq). In addition, one patient had triple X syndrome, and two patients were found to carry FMR1 premutations. One further case involved congenital ovarian agenesis. 

Among patients with idiopathic POI, 9 out of 41 (22%) had a positive family history, while familial predisposition was also observed in 2 additional cases—one iatrogenic and one autoimmune. In none of these cases was an FMR1 premutation or chromosomal abnormality confirmed.

**Figure 4:**
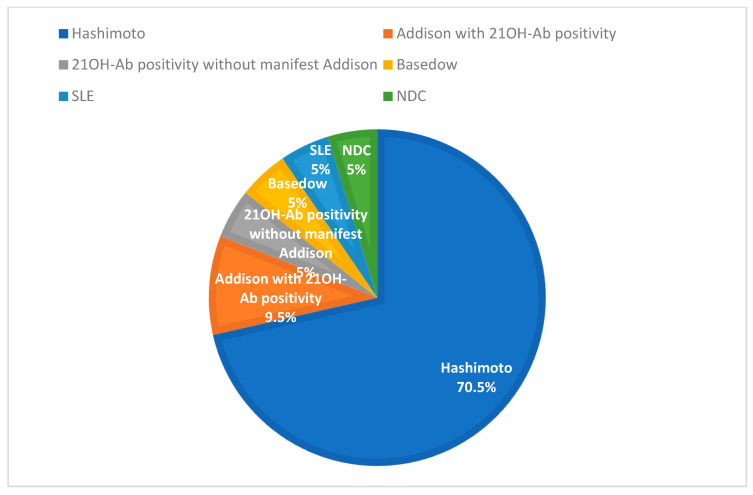
Distribution of autoimmune POI etiologies in the contemporary cohort 2017–2024; n = 21).

**Figure 5:**
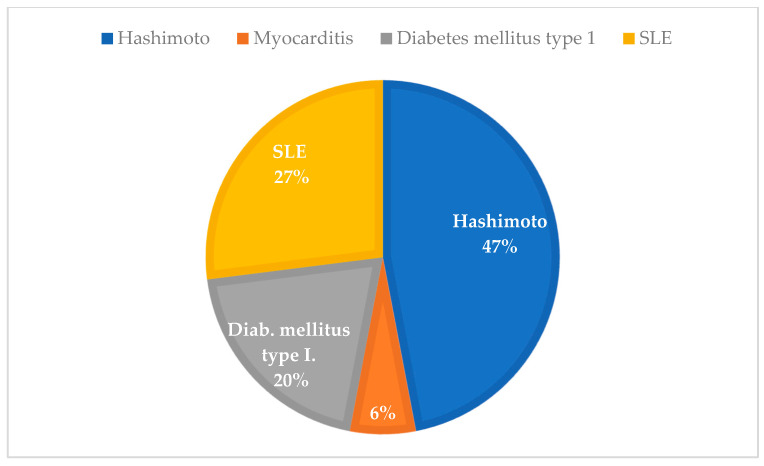
Distribution of autoimmune POI etiologies in the historical cohort, 1978–2003; (n = 15).

Of the 21 patients with autoimmune-related POI in the contemporary cohort, 15 were associated with Hashimoto’s thyroiditis, 2 with Addison’s disease and 21OH-Ab positivity, 1 with isolated 21OH-Ab positivity without manifest Addison, 1 with Graves’ disease, 1 with SLE, and 1 with non-specified connective tissue diseases (NDC) (Figure 4). In the historical cohort, autoimmune etiologies included 7 cases of Hashimoto’s thyroiditis, 4 cases of SLE, 3 cases of type 1 diabetes mellitus, and 1 case of chronic myocarditis—potentially indicative of polyglandular autoimmune syndrome (PAS) (Figure 5).

**Figure 6:**
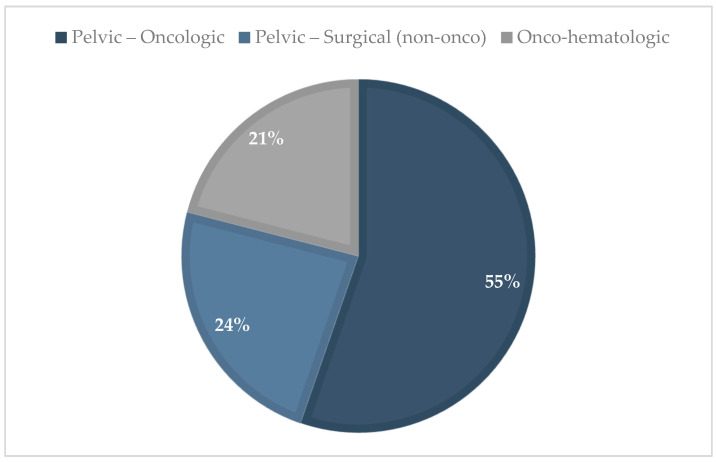
Distribution of iatrogenic POI etiologies in the contemporary cohort, 2017–2024; (n = 38).

Among the 38 patients with iatrogenic POI, 29 cases (76%) were attributed to oncologic causes. These included 8 patients treated for hematologic malignancies and 20 patients who underwent extensive surgery and adjuvant therapies for pelvic malignancies—primarily of gynecologic origin—and one case related to breast cancer. The remaining 9 cases (24%) were associated with non-oncologic surgical interventions (Figure 6). Of these, 5 procedures were performed due to endometriosis, while other indications included ovarian cystectomy (2 cases), adnexectomy (1 cases), and uterine artery embolization (1 case).

**Figure 7:**
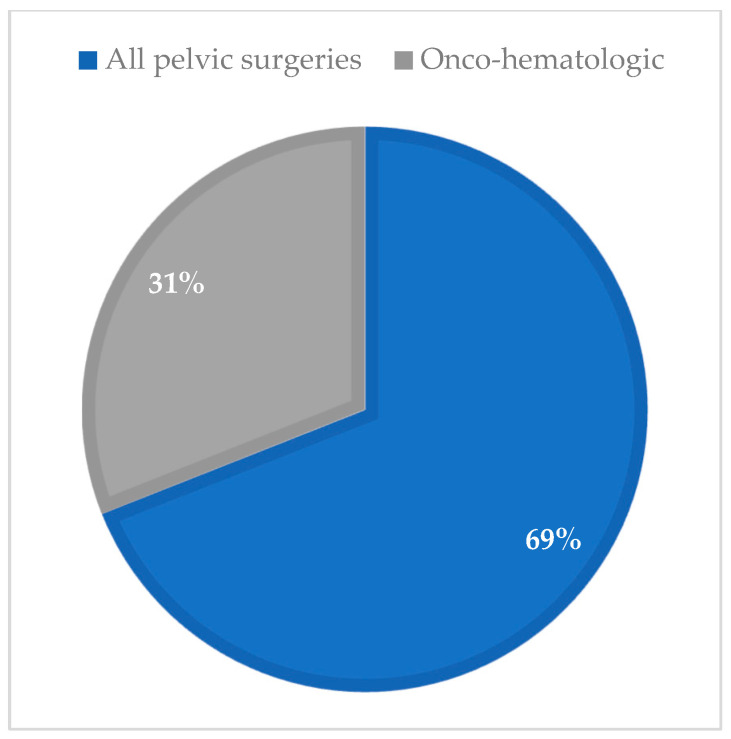
Distribution of iatrogenic POI etiologies in the historical cohort, 1978–2003; (n =13).

The comparison of iatrogenic POI etiologies between cohorts reveals a notable shift over time. In the historical cohort, pelvic surgeries and hematologic causes accounted for 69% and 31% of iatrogenic cases, respectively. In the contemporary cohort, pelvic surgeries increased to a total of 79% (comprising 55.3% oncologic and 23.7% surgical), while hematologic causes were slightly lower at 21% (Figure 7). Notably, among the surgical cases in the contemporary cohort, more than half (55%) were associated with endometriosis-related procedures.

### 3.5. Reproductive Outcomes in the Two Groups

In terms of reproductive history, a total of ten pregnancies were reported among POI patients following diagnosis. Two pregnancies occurred spontaneously, while eight were achieved through in vitro fertilization (IVF), including two with confirmed use of oocyte donation. Among the IVF pregnancies, three ended in miscarriage, one of which was medically induced due to fetal malformation. In addition, 13 patients underwent unsuccessful fertility consultations or treatments at assisted reproduction centers (ARCs). In our cohort of 111 women with POI, the live birth rate was 6.3% (7/111), reflecting the persistently limited reproductive success in this patient population despite advances in reproductive medicine.

## 4. Discussion

This study was designed to examine the evolving distribution of POI etiologies over recent decades, with the aim of highlighting the increasing proportion of iatrogenic cases and drawing attention to modifiable clinical practices. By comparing two cohorts from the same center, we sought to identify trends that may inform preventive strategies and guide more personalized approaches to preserve fertility, optimize health outcomes, and improve the quality of life in women affected by premature ovarian insufficiency.

Between 1978 and 2003, a total of 172 women were diagnosed with POI at our center. In contrast, 111 patients were diagnosed over just eight years (2017–2024), indicating a higher number of POI cases within a considerably shorter timeframe. This reflects an approximately 2.1-fold increase in the annual rate of new POI diagnoses in the contemporary cohort compared to the historical one.

Interestingly, no significant change was observed in the distribution of primary versus secondary amenorrhea, nor in the proportion of genetically confirmed cases between the two cohorts. This may reflect limitations in access to comprehensive genetic testing and the fact that many monogenic forms of POI remain undetected. Karyotyping and *FMR1* premutation analysis are currently recommended for all patients diagnosed with POI, regardless of age, while the routine use of next-generation sequencing remains limited due to cost and lack of direct therapeutic implications [1]. Although WGS has revealed both X-linked and autosomal mutations implicated in POI [3], most genetic contributors remain unidentified, underscoring the complex and heterogeneous genetic architecture of the condition [17,54].

Our findings demonstrate a marked shift in the distribution of POI etiologies over the past decades. While idiopathic cases accounted for 72% in the earlier cohort (1978–2003), their proportion decreased to 37.6% in the contemporary cohort (2017–2024). The predominance of idiopathic POI in our cohort may reflect underlying, yet unrecognized genetic causes—an assumption further supported by the observation that 22% of idiopathic cases showed familial clustering, highlighting the potential role of heritable factors and the need for more comprehensive genetic screening in this subgroup.

Our results align with those of a 2022 Finnish population-based study, which reported a 4.6-fold increased risk of POI among first-degree relatives of affected individuals compared to controls [55]. Similarly, a 2023 multigenerational genealogical study identified familial links in 6.3% of POI cases when considering first-, second-, and third-degree relatives [56].These findings confirm the existence of familial clustering, although with lower prevalence estimates than those reported in earlier studies, such as the 31% prevalence based on self-reported family histories [57,58,59]. While these literature-based estimates are numerically lower than the 22% observed in our idiopathic subgroup, they are not directly comparable due to methodological differences. Our figure reflects familial clustering specifically within idiopathic cases, based on retrospective self-reported family history, whereas the cited studies evaluated the entire POI population using broader or more systematic approaches.

Nevertheless, all findings consistently underscore the relevance of familial screening and support the hypothesis that a proportion of idiopathic POI cases may represent undiagnosed heritable forms. These observations reinforce the value of incorporating detailed family history assessments and expanded genetic testing into the diagnostic workup of idiopathic POI.

In our cohort, autoimmune causes accounted for 18.9% of all POI cases, which is consistent with recent findings from a large study involving 610 women with POI, in which at least one autoimmune disease was identified in 25% of patients [20]. Registry-based data from Finland similarly demonstrated that 5.6% of women with POI had a severe autoimmune disease prior to diagnosis, and that the risk of developing a new autoimmune condition was almost threefold higher during the first three years after POI onset (OR = 2.6) [60].

Polyglandular autoimmune syndromes (PAS), particularly types I and II, have been strongly implicated in the pathogenesis of POI. Type I PAS, caused by mutations in the AIRE gene, often includes POI as part of its clinical spectrum, alongside Addison’s disease, hypoparathyroidism. Type II PAS, while more common, involves POI less frequently [3,60]. Notably, PAS has been associated with a dramatically increased POI risk, with an odds ratio (OR) of 25.8 compared to controls [60].

Additionally, up to 10% of women with autoimmune Addison’s disease are reported to develop POI, often preceding or coinciding with adrenal insufficiency [61]. This observation is consistent with our findings, as 10% of autoimmune POI cases in our cohort were also associated with Addison’s disease, supporting the close pathophysiological link between the two conditions.

Despite these associations, current treatment options for autoimmune POI remain limited. Although corticosteroids have been historically applied, their clinical benefit appears restricted to select cases, and no immunotherapy has been robustly validated for routine use [18]. Recent experimental studies, however, offer a potential therapeutic direction: in an animal model of autoimmune POI, hydroxychloroquine improved ovarian function by modulating immune balance through Treg/Th17 regulation and reducing ovarian inflammation, partially rescuing ovulatory function [62]. These preliminary findings warrant further investigation in human studies.

Our findings demonstrate a significant rise in the prevalence of iatrogenic POI, which accounted for 34.2% of cases in the contemporary cohort, compared to only 7.6% in the historical cohort. Several factors may contribute to this shift. The growing use of surgical interventions, particularly ovarian surgeries for endometriosis, has likely played a role. Additionally, advances in oncology and increased survival rates among young women have led to a higher number of patients exposed to gonadotoxic treatments such as chemotherapy and radiotherapy. This is consistent with recent population-based data showing that adolescent and young adult (AYA) cancer survivors have a significantly elevated risk of developing POI compared to women without a history of cancer (5.4% vs. 2.2%), with an overall adjusted relative risk (aRR) of 2.49 [63]. These findings underscore the importance of incorporating fertility-preserving strategies into the planning of gynecologic and oncologic treatments, particularly for women of reproductive age. Pre-treatment counseling and individualized reproductive planning should be considered standard components of care.

### 4.1. How Can We Improve Outcomes in Women Diagnosed with POI, Particularly Those with Iatrogenic Etiology?

In iatrogenic cases, prevention is key, encompassing a range of fertility-preserving strategies later detailed in this section—including judicious use of gonadotoxic therapies, ovarian transposition (OT), and intraoperative protection of the ovarian reserve. In women undergoing oncologic treatment, the use of GnRH agonists like goserelin may help preserve ovarian function in selected populations. A recent systematic review demonstrated that the addition of goserelin to chemotherapy in women with early-stage breast cancer significantly reduced the risk of developing POI, supporting its use as a fertility-preserving strategy in selected patients. Nevertheless, it is important to emphasize that goserelin does not replace cryopreservation techniques, such as oocyte or embryo freezing, which remain the gold standard for fertility preservation [64]. Additionally, a recent study introduced a machine learning–based model with 88% accuracy to predict individual risk of POI after chemotherapy, offering a promising tool for personalized oncofertility counseling [65].

According to a systematic review, OT should be considered in the management of premenopausal women with cervical cancer, as it is associated with a high rate of ovarian function preservation and no reported cases of metastasis to the transposed ovaries—potentially preventing iatrogenic menopause or POI [45,66].

In surgical cases, especially those involving endometriosis, individualized treatment strategies and long-term follow-up are essential. Prior to surgical intervention for endometrioma, careful consideration should be given to the patient’s age, baseline anti-Müllerian hormone (AMH) level, and future reproductive plans [47]. Intraoperatively, when hemostasis is required, oxidized cellulose polymers and suturing techniques should be preferred over bipolar coagulation, as these methods have been shown to better preserve ovarian reserves [67,68]. Additionally, performing laparoscopic cystectomy during the luteal phase may further minimize surgical damage to ovarian tissue [69]. More broadly, benign gynecologic surgeries—such as hysterectomy or salpingectomy—may impair ovarian function even when the ovaries are preserved, potentially accelerating ovarian aging [70]. TLH has been associated with an earlier onset of menopause by an average of 3.7 years [71]. Bilateral salpingo-oophorectomy (BSO), often performed as a preventive measure—particularly in BRCA1/2 carriers—offers survival benefits but necessitates individualized hormone replacement therapy (HRT) [72]. It is essential that patients are adequately counseled on whether definitive surgical management is truly indicated for benign symptoms such as abnormal uterine bleeding, and on the importance and availability of HRT following surgery.

While hormone replacement therapy (HRT) remains the cornerstone of POI management, several experimental strategies are under investigation. In vitro follicle activation *(IVA)* targets dormant follicles via key signaling pathways. Stem cell and exosome therapies show regenerative potential but lack strong clinical validation. Intraovarian PRP injections have induced spontaneous ovulation in selected cases. Other emerging options include mitochondrial activation and miRNA-based therapies, currently in preclinical phases [73].

### 4.2. Factors Contributing to Increased Detection and Etiological Reclassification

The observed increase in the detection rate of POI in the contemporary cohort may reflect improved clinical awareness, greater access to hormonal assays, and evolving diagnostic criteria. Importantly, patient awareness has also improved, with more women seeking medical evaluation for menstrual irregularities and fertility concerns at earlier stages. In addition, the FSH threshold used to define POI has been lowered from 40 IU/L to 30 IU/L in recent guidelines, allowing for earlier and more frequent diagnoses [2,51].

Furthermore, routine autoimmune antibody testing has become part of the diagnostic panel in recent years, facilitating more precise classification of autoimmune-related cases. In contrast, in the historical cohort, such assays were not available, and autoimmune thyroiditis was typically diagnosed via fine-needle aspiration cytology of the thyroid demonstrating lymphocytic infiltration. Evolving diagnostic practices and the availability of autoantibody testing may therefore have influenced cohort-level differences in autoimmune POI classification and recognition.

Alongside improved detection, a shift in the distribution of POI etiologies has also been observed. The relative proportion of iatrogenic POI has increased considerably, likely driven by the growing number of oncologic and gynecologic interventions in reproductive-aged women. Importantly, the success of cancer treatments has resulted in more long-term survivors who may experience treatment-induced gonadotoxicity. The degree of gonadotoxicity varies across chemotherapeutic regimens and surgical approaches; however, due to the retrospective design and incomplete intraoperative documentation, more detailed stratification of treatment-related risk was not feasible in our study. Environmental factors and the increasing recognition of autoimmune disorders may also contribute to the rise in non-idiopathic cases. As a consequence, idiopathic POI now represents a smaller proportion of all diagnosed cases—not necessarily due to a decrease in absolute numbers, but rather due to the expansion of identified etiologies through more precise diagnostic tools and evolving clinical patterns.

### 4.3. Reproductive Outcome

Despite advances in reproductive medicine over the past decades, reproductive outcomes among women with POI remain suboptimal. In the historical cohort from 1978 to 2003, ten pregnancies were recorded, resulting in six live births, three miscarriages, and one ectopic pregnancy. In our contemporary cohort, a comparable total of ten pregnancies occurred, with seven resulting in live births. These data suggest that while assisted reproductive technologies are more widely available today, successful reproductive outcomes in POI patients remain limited. Our cohort’s live birth rate of 6.3% is consistent with previously published data. In one study, 7.7% of women with POI had a live birth, and 8.6% experienced spontaneous pregnancy [74]. Another report found a 5.8% live birth rate in POI patients undergoing IVF with embryo transfer [75]—placing our result within the expected range across both spontaneous and assisted reproductive outcomes.

In terms of fertility, while options remain limited, early fertility counseling, cryopreservation prior to treatment, and access to assisted reproductive technologies—including oocyte donation—should be considered. Although novel therapies such as stem cell transplantation and immunomodulation are under investigation, their clinical applicability remains uncertain. Thus, multidisciplinary care and individualized reproductive planning remain central to optimizing health and quality of life in women with POI [3].

### 4.4. Strengths and Limitations

The major strength of this study is its cohort design spanning multiple decades within a single center, allowing for consistent diagnostic methodology and data retrieval. The inclusion of both historical and contemporary data strengthens the internal validity of observed trends. However, limitations include the retrospective design, incomplete data in some archived files, and differences in diagnostic criteria (e.g., FSH threshold changes) that may partially account for the shift in etiologic classifications. Furthermore, our diagnostic approach to autoimmune POI was relatively broad, as it included Hashimoto’s thyroiditis with anti-TPO positivity. Although this followed the guidelines available at the time (ESHRE 2016, NICE 2019) [2,52], thyroid autoimmunity is common and not always causally linked to POI, which may have led to overestimation of autoimmune cases. In line with the latest ESHRE recommendations (2024) [1], future studies should use stricter criteria and prioritize adrenal cortex antibody (21OH-Ab) testing to improve specificity.

## 5. Conclusions

While etiology-directed therapy is currently not feasible in most POI cases, its underlying causes remain essential in identifying potentially preventable risk factors and informing targeted prevention strategies. Our findings support early diagnosis, individualized fertility preservation strategies, and heightened awareness of iatrogenic risks. These findings underscore the importance of future prospective follow-up studies to enable more detailed stratification of iatrogenic cases, including specific chemotherapeutic regimens and surgical techniques, in order to better understand their distinct gonadotoxic impacts and to inform individualized fertility preservation approaches. Future research should focus on improving genetic diagnostics, validating immunomodulatory therapies, and strengthening multidisciplinary care pathways to optimize reproductive and general health in women with POI.

## Figures and Tables

**Table 1:** Age, BMI, and laboratory data of patients in Group 1.

	All POI Patients (n = 111)	Genetic POI (n = 11)	Autoimmune POI (n = 21)	Iatrogenic POI (n = 38)	Idiopathic POI (n = 41)
Age at diagnosis (years ± SD)	32.85 ± 8.81	**19.7** ± **7.33**	31.55 ± 29.16	35.11 ± 8.49	34.96 ± 6.44
BMI (kg/m^2^ ± SD)	25.5 ± 5.82	24.42 ± 8.13	25.37 ± 3.67	26.01 ± 6.48	25.33 ± 5.54
FSH (IU/L ± SD)	83.66 ± 42.25	66.66 ± 22.13	70.05 ± 29.16	93.92 ± 44.73	87.37 ± 47.75
LH (IU/L ± SD)	44.75 ± 18.77	35.8 ± 11.26	41.94 ± 17.3	49.03 ± 19.99	45.28 ± 19.65
E2 (ng/L ± SD)	26.35 ± 35.91	30.15 ± 37.55	32.7 ± 36.95	**12.52** ± **16.16**	33.88 ± 44.16
TSH (mIU/L ± SD)	2 ± 1.5	1.5 ± 0.56	2.04 ± 1.42	2.18 ± 2.11	1.96 ± 0.97
PRL (μg/L ± SD)	10.55 ± 6.1	12.27 ± 9.4	9.65 ± 5.25	11.98 ± 7.69	9.7 ± 4.74

Note: Bolded values represent statistically significant difference between data points within the same row (*p* < 0.05).

## Data Availability

The data presented in this study are available by contacting the corresponding author.
